# Metapopulation dynamics and foraging plasticity in a highly vagile seabird, the southern rockhopper penguin

**DOI:** 10.1002/ece3.6127

**Published:** 2020-03-05

**Authors:** Nicolás A. Lois, Leonardo Campagna, Ulises Balza, Michael J. Polito, Klemens Pütz, Juliana A. Vianna, Annick Morgenthaler, Esteban Frere, Ricardo Sáenz‐Samaniego, Andrea Raya Rey, Bettina Mahler

**Affiliations:** ^1^ Instituto de Ecología, Genética y Evolución de Buenos Aires Consejo Nacional de Investigaciones Científicas y Técnicas (IEGEBA‐CONICET) Buenos Aires Argentina; ^2^ Departamento de Ecología, Genética y Evolución Facultad de Ciencias Exactas y Naturales Universidad de Buenos Aires (DEGE‐FCEyN‐UBA) Buenos Aires Argentina; ^3^ Centro Austral de Investigaciones Científicas Consejo Nacional de Investigaciones Científicas y Técnicas (CADIC‐CONICET) Ushuaia Argentina; ^4^ Fuller Evolutionary Biology Program Cornell Lab of Ornithology Cornell University Ithaca NY USA; ^5^ Department of Ecology and Evolutionary Biology Cornell University Ithaca NY USA; ^6^ Department of Oceanography and Coastal Sciences Louisiana State University Baton Rouge LA USA; ^7^ Antarctic Research Trust Bremervörde Germany; ^8^ Departamento de Ecosistemas y Medio Ambiente Facultad de Agronomía e Ingeniería Forestal Pontificia Universidad Católica de Chile Santiago Chile; ^9^ Centro de Investigaciones Puerto Deseado UACO Universidad Nacional de la Patagonia Austral Puerto Deseado, Santa Cruz Argentina; ^10^ Instituto de Ciencias Polares Ambiente y Recursos Naturales Universidad Nacional de Tierra del Fuego (ICPA‐UNTdF) Ushuaia Argentina; ^11^ Wildlife Conservation Society Buenos Aires Argentina

**Keywords:** ddRAD, *Eudyptes chrysocome*, population dynamics, SIBER, trophic niche

## Abstract

Population connectivity is driven by individual dispersal potential and modulated by natal philopatry. In seabirds, high vagility facilitates dispersal yet philopatry is also common, with foraging area overlap often correlated with population connectivity. We assess the interplay between these processes by studying past and current connectivity and foraging niche overlap among southern rockhopper penguin colonies of the coast of southern South America using genomic and stable isotope analyses. We found two distinct genetic clusters and detected low admixture between northern and southern colonies. Stable isotope analysis indicated niche variability between colonies, with Malvinas/Falklands colonies encompassing the species entire isotopic foraging niche, while the remaining colonies had smaller, nonoverlapping niches. A recently founded colony in continental Patagonia differed in isotopic niche width and position with Malvinas/Falklands colonies, its genetically identified founder population, suggesting the exploitation of novel foraging areas and/or prey items. Additionally, dispersing individuals found dead across the Patagonian shore in an unusual mortality event were also assigned to the northern cluster, suggesting northern individuals reach southern localities, but do not breed in these colonies. Facilitated by variability in foraging strategies, and especially during unfavorable conditions, the number of dispersing individuals may increase and enhance the probability of founding new colonies. Metapopulation demographic dynamics in seabirds should account for interannual variability in dispersal behavior and pay special attention to extreme climatic events, classically related to negative effects on population trends.

## Graphical abstract

With the use of genomic and stable isotopes techniques, we provide new insights on the relationship between the use of different foraging grounds and dispersal‐driven population connectivity in a seabird species. We discover two recently isolated lineages within this species presenting different foraging and dispersal behavior.

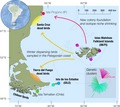

## 1 BACKGROUND

Gene flow homogenizes populations, while barriers favor differentiation and, over time, the development of evolutionary independent lineages. Population connectivity is crucial to understand the dynamics of fragmented populations (Bicknell et al., 2014). An extreme case of fragmentation occurs in seabirds due to specific life‐history traits: long foraging trips and high vagility, together with discrete reproductive patches (i.e., colonies) and high levels of philopatry. These counteracting life‐history traits have been described as the seabird paradox (Milot, Weimerskirch, & Bernatchez, 2008). When dispersal (i.e., the movement between birth and breeding sites) is not physically constrained, nonphysical barriers become more evident, making highly mobile seabird species great models to study these types of restrictions to gene flow (Friesen, 2015). However, the processes that regulate seabird population connectivity and the foundation of new colonies are often challenging to explore.

Multiple hypotheses have been proposed to explain restricted gene flow in the absence of physical barriers (Friesen, 2015; Munro & Burg, 2017), the leading hypothesis being philopatry, a well‐documented life‐history trait in seabirds (Coulson, 2002). Nonoverlapping foraging grounds have been proposed as another factor limiting gene flow (Friesen, 2015). During the breeding season and prior to molting, seabirds act as central place foragers, attempting to maximize energy intake by foraging as close as possible to their colony, which drives high intraspecific competition and niche partitioning (Orians & Pearson, 1979). The resulting degree of foraging overlap has been found to correlate with population connectivity, that is, species with population‐specific foraging grounds show higher genetic structure (Burg & Croxall, 2001; Calderón, Quintana, Cabanne, Lougheed, & Tubaro, 2014), even when breeding in nearby colonies (Lombal et al., 2017). In these cases, the opportunity for gene flow is much lower than in those that have a common nonbreeding area. Stable isotope analysis complements studies of gene flow and population connectivity (Rayner et al., 2011) as it has become a common tool to assess the movements, diets, and foraging overlap of seabirds (Ramos & González‐Solís, 2012).

The crested penguins (*Eudyptes* spp.) are a complex of threatened species (IUCN, 2019), which inhabit between 30 and 60°S worldwide and breed from September to March in temperate and subantarctic islands (Pütz, Raya Rey, & Otley, 2013). Their taxonomic status is still debated and oceanographic fronts have been suggested as the main driver for their divergence at a global scale (Frugone et al., 2018). However, whether finer‐scale oceanographic conditions or behavioral traits could also drive population structure remains to be studied. Like all seabirds, they are suspected to be predominantly philopatric, showing fidelity to their breeding sites (Coulson, 2002) and also segregation within their foraging areas (Thiebot, Cherel, Trathan, & Bost, 2012). During their wintering at sea, satellite‐tracked individuals have shown these species travel between 2,000 and 8,000 km (Bost, Thiebot, Pinaud, Cherel, & Trathan, 2009; Pütz, Raya Rey, Schiavini, Clausen, & Lüthi, 2006; J. Thiebot, Cherel, Trathan, & Bost, 2011), evidencing their high dispersal potential.

In light of an always increasing anthropogenic pressure and the current climate change scenario, information on seabird species movements and plasticity to environmental changes are critical to understand their short‐ and long‐term viability (Cristofari et al., 2019; Munro & Burg, 2017). Here, we analyze regional genetic divergence among colonies of the southern rockhopper penguin (hereafter ‘rockhopper’, *E. chrysocome*) off the coast of southern South America and explore their foraging niche through stable isotope analysis to study regional population connectivity. Previous evidence shows that *Eudyptes* are plastic foragers both during the breeding (Horswill, Trathan, & Ratcliffe, 2017) and nonbreeding season (Pütz et al., 2006), which buffers the species from variation in interannual food availability (Dehnhard et al., 2016), while they possess population‐specific winter foraging distribution with little overlap (Pütz et al., 2006; Ratcliffe et al., 2014). Therefore, we assess whether intraspecific population structure is related to colony‐specific foraging niche (i.e., foraging areas and/or diets). Finally, we leverage a recently founded (ca. 1980) colony in Isla Pingüino near the South American continent (Gandini, Millones, Morgenthaler, & Frere, 2016) and a huge mortality event that occurred throughout the Patagonian Atlantic coast in 2016 during the nonreproductive period (Morgenthaler et al., 2018), to provide insights into the dispersal behavior of this species.

## 2 METHODS

### 2.1 Study sites and sampling

**Figure 1:**
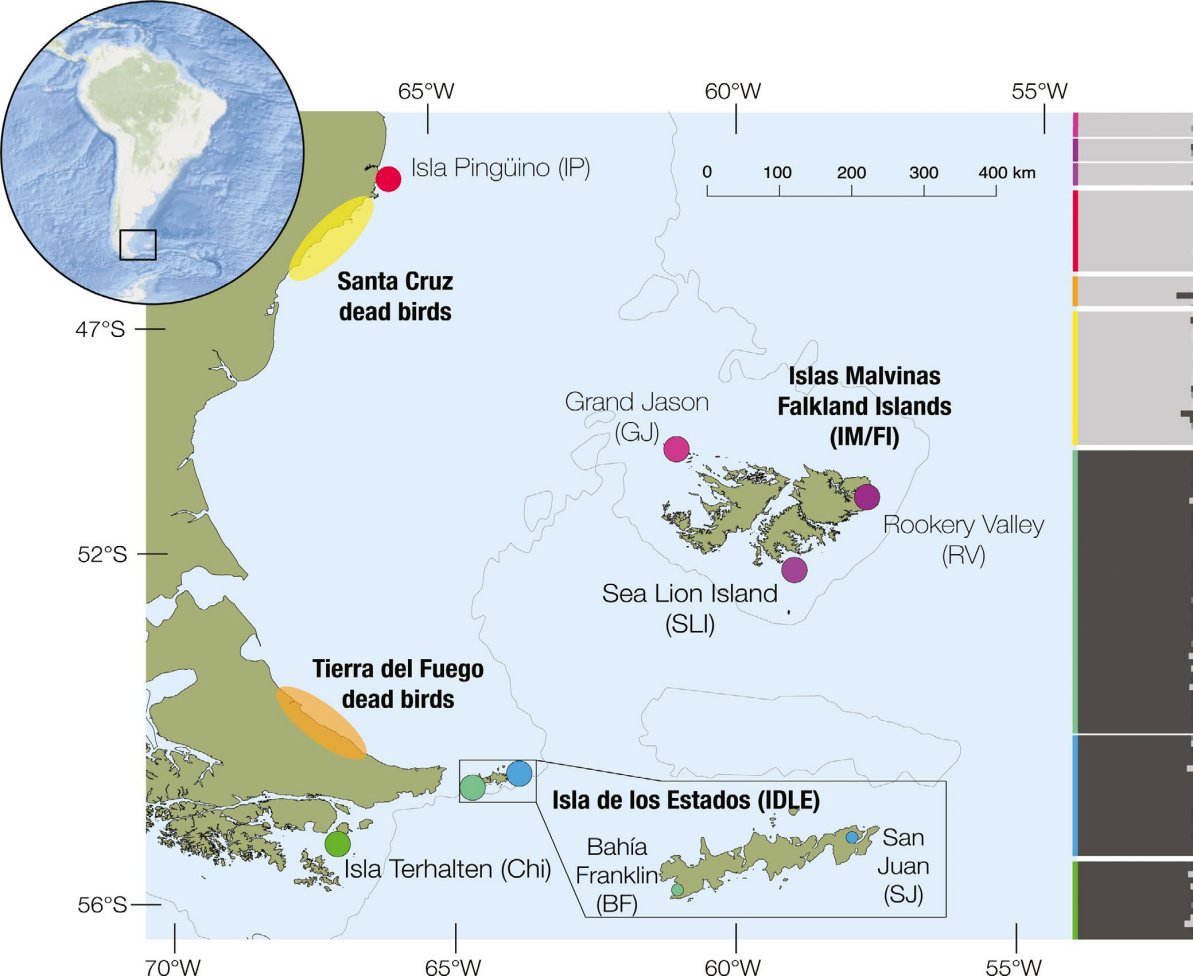
Map showing the study area within *E. chrysocome* distribution. Dots indicate sampled colonies and shaded areas along the coastline indicate sampling areas of stranded individuals. Population structure is shown to the right in the vertical STRUCTURE plot (*K* = 2, color‐coded as colonies)

A total of 209 *E. chrysocome* individuals were sampled at seven different colonies within the Patagonian shelf in the Southwestern Atlantic Ocean (SWAO) (Figure 1): Terhalten, Chile (Chi); San Juan de Salvamento (SJ) and Bahía Franklin (BF), both in Isla de los Estados (IDLE), Argentina; Rookery Valley (RV), Grand Jason (GJ), and Sea Lion Island (SLI), all three in Islas Malvinas/Falkland Islands (IM/FI); and a small, recently founded (ca. 1980) colony in Isla Pingüino (IP), Santa Cruz, Argentina. Also, 34 wintering dead birds were sampled along the Patagonian eastern coast (from 47.09°S, 65.87°W to 53.78°S, 67.7°W) during autumn and winter of 2016 (Figure 1).

### 2.2 DNA sequencing

We purified genomic DNA blood or tissue samples from all study sites using Gentra extraction kit (Qiagen) and generated double‐digest restriction‐site associated DNA markers (ddRADtags) for the 160 samples that had the best quality DNA (Table S1) following Thrasher, Butcher, Campagna, Webster, and Lovette (2018) workflow. In short, genomic DNA was digested using two restriction enzymes, SbfI and MspI, and the resulting fragments were ligated to P1 (with a unique barcode) and P2 (with an index group barcode) adapters to the 5’ and 3’ ends, respectively. We pooled 160 samples in eight groups, each sample containing a unique combination of barcodes for posterior demultiplexing. We size‐selected DNA libraries using BluePippin (Sage Science), retaining fragments between 400 and 700 bp, and finally amplified the DNA fragments by performing 9 PCR cycles with Phusion DNA Polymerase (NEB). After cleanup with AMPure beads, we pooled the 8 groups in equimolar ratios to create a single library for sequencing. The final library containing 160 individuals was sequenced on one lane of an Illumina HiSeq 2500 at the Cornell University Biotechnology Resource Center, producing 221.4 million 101 bp single‐end reads.

### 2.3 Bioinformatics and assembly of RAD loci

The quality of the reads was assessed using FastQC version 0.11.6 (http://www.bioinformat-ics.babraham.ac.uk/projects/fastqc). To exclude low quality calls, we trimmed sequences to 97 bp using fastX_trimmer (Gordon & Hannon, 2010). All reads containing any base with a Phred quality score below 10 (90% call accuracy) or with more than 5% between 10 and 20 (99% call accuracy) were removed using fastq_quality_filter (fastx‐Toolkit). We demultiplexed the reads using the process_radtags module from the stacks pipeline version 1.44 (Catchen, Hohenlohe, Bassham, Amores, & Cresko, 2013) to obtain files containing sequences that were specific to each individual.

Individual reads were then aligned to the Adélie penguin (*Pygoscelis adeliae*) reference genome version GCA_000699105.1, obtained from http://www.ncbi.nlm.nih.gov, using Bowtie2 version 2.2.8 (Langmead & Slazberg, 2012), and producing an average alignment rate of 79.7%. We assembled the aligned sequences into RAD loci using the ref_map.pl script from the Stacks pipeline (Catchen et al., 2013). We ran the modules of the pipeline separately: ustacks–cstacks–sstacks, followed by the error correction model rxstacks, and finally another iteration of cstacks–sstacks. SNPs were then exported using the populations module, retaining loci present in at least 80% of the individuals (r parameter) and at a minimum stack depth of 10 (m parameter). We applied a minor allele frequency filter of at least 0.01 (‐‐min_maf) and exported the remaining SNPs in different datasets as variant call (.vcf) and structure (.str) formats (see details on the individuals included in Table S1 and a summary of the number of SNPs and loci analyzed in each dataset in Table S2). Summary statistics on genetic diversity per colony and archipelago (i.e., colonies within the same group of islands) were calculated in poppr (Kamvar, Brooks, & Grünwald, 2015) in R environment (R Core Team, 2018).

### 2.4 Population genomic analyses

We calculated diversity (*H*, *H*_exp,_ and *H*_obs_) and inbreeding (*F*_IS_ and kinship) statistics for the seven colonies (Table 1) and assessed the overall level of differentiation calculating individual (per locus) and general (all loci) SNP (*F*_ST_) and haplotype (*Φ*_ST_) differentiation between each pair of populations with Stacks. We also performed an AMOVA‐based *F*_ST_ estimation with poppr package in R environment using 1,000 permutations to test for significance with three levels of spatial clustering: colonies, archipelagos, and northern–southern colonies.

**Table 1:** Summary statistics and sample sizes by sampling site

Site/ Colony	Code	*n*	*H*	*H*_exp_	*H*_obs_	*F*_IS_	Kinship
NORTH		26	3.26	0.194	0.186	0.042	0.029
Continental Patagonia		14					
Isla Pingüino	IP	14	2.64	0.191	0.183	0.033	
Malvinas/Falklands	IM/FI	12	2.48	0.196	0.188	0.037	
Grand Jason	GJ	4					
Rookery Valley	RV	4					
Sea Lion Island	SLI	4					
SOUTH		81	4.39	0.210	0.201	0.047	0.0067
Southern Chile		13					
Isla Terhalten	Chi	13	2.56	0.207	0.198	0.030	
Isla de los Estados	IDLE	68					
Bahía Franklin	BF	48	3.87	0.211	0.202	0.047	
San Juan	SJ	20	3.00	0.209	0.202	0.030	
Total		107	4.67	0.201	0.203	0.041	0.013

Note

We present Shannon–Weiner diversity index (*H*), Nei's gene diversity, expected heterozygosity (*H*_exp_), observed heterozygosity (*H*_obs_), inbreeding coefficient (*F*_IS_) for each colony (except for IM/FI colonies due to small sample size), and the average kinship within northern and southern colonies and among all individuals.

We used three clustering methods to analyze our SNP data: principal component analysis (PCA) using the SNPrelate R package, the Bayesian clustering algorithms in STRUCTURE (Pritchard, Stephens, & Donnelly, 2000 ), and fineRADstructure v 0.2 (Malinsky, Trucchi, Lawson, & Falush, 2018). STRUCTURE estimates membership coefficients of each individual to each of the inferred genetic clusters (*K*) using independent SNPs (the first SNP of each locus, see Table S2). We implemented the admixture ancestry model and used correlated allele frequencies. In order to estimate the allele frequency prior value (lambda), we set *K* = 1 and ran the model for 750,000 generations, discarding the first 250,000 as burn‐in, allowing lambda to vary. We then ran the program for the same number of generations (discarding the initial burn‐in of 250,000), using this lambda value and *K* = 1 through 4 (conducting 10 iterations per K value with a different random seed per iteration). We used structure harvester v0.6.94 (Earl & von Holdt, 2012) and clumpp v1.1.2 (Jakobsson & Rosenberg, 2007) to combine results from replicate runs and calculate the average membership coefficients of each individual to each cluster. We report likelihood values for different values of K and discuss their biological relevance following Meirmans (2015) without applying the Evanno method for selecting the optimal K, as it has been shown to perform poorly in scenarios where there is low genetic differentiation (Waples & Gaggiotti, 2006). We compared the STRUCTURE results with those from fineRADstructure v 0.2 (Malinsky et al., 2018), which infers a co‐ancestry matrix of haplotypes through a Markov chain Monte Carlo clustering algorithm and quantifies nearest neighbor relationships between all individuals in the analysis. Finally, we conducted a phylogenetic analysis using SNP data in SVD quartets (Chifman & Kubatko, 2014).

For demographic modeling, we used the topology inferred in SVD quartets and co‐estimated divergence times, effective population sizes, and gene flow using the Generalized Phylogenetic Coalescent Sampler (G‐PhoCS) version 1.2.2 (Gronau, Hubisz, Gulko, Danko, & Siepel, 2011). Because of the computationally intensive nature of this analysis, we subsampled the dataset and only included 26 individuals from the IM/FI–IP group (see below) and 59 individuals from the Chi–IDLE group, using only one haplotype per individual (subsampling the Chi–IDLE group to 26 individuals and using both haplotypes per individual did not change our findings significantly). We re‐exported loci for these 85 individuals and used sequence data from 4,453 variant and invariant RAD loci in our model which included six free parameters: two current and one ancestral effective population size, and one divergence time and two migration rates. G‐PhoCS was run for 450,000 generations using the default settings for the Markov chain Monte Carlo and discarding the initial 50,000 generations as burn‐in. We inspected the convergence of the model in Tracer 1.7 (Rambaut, Drummond, Xie, Baele, & Suchard, 2018). We used an approximate mutation rate of 10^–9^ mutations per bp per generation (Smeds, Qvarnström, & Ellegren, 2016) to convert from mutation scale to generations. However, since mutation rates are known to highly vary between species and genomic regions, we focus mainly on relative comparisons, which are not impacted by the assumption of a general mutation rate. We measured gene flow in migrants per generation, which we calculated as the per generation migration rate times a fourth of the theta parameter for the receiving population:

$$m_{a>b}\;\times \;\theta_{b}/4.$$

### 2.5 Stable isotope analysis

Stable isotope analysis is a noninvasive and cost‐effective technique that can be used as a proxy of the foraging behavior of a population. Carbon stable isotope values (δ^13^C) reflect basal carbon sources within a food web and can be used to trace marine habitat use by consumers (Cherel & Hobson, 2007). In addition, nitrogen stable isotope values (δ^15^N) reflect the trophic position of consumers due to a step‐wise enrichment of δ^15^N values between trophic levels (Minagawa & Wada, 1984). Furthermore, spatial variation in δ^13^C and δ^15^N values at the base of marine food webs has been commonly used to identify geographic foraging area of marine consumers (Cherel & Hobson, 2007; Graham, Koch, Newsome, McMahon, & Aurioles, 2010). Because of their natural covariance the δ^15^N and δ^13^C values are commonly presented as a biplot and act to delineate a population of consumers’ “isotopic niche.” For example, when two populations forage in the same area and/or exploit the same resources, they are expected to have overlapping δ^15^N and δ^13^C values and thus overlapping isotopic foraging niches (Newsome, Martinez del Río, Bearhop, & Phillips, 2007).

We analyzed feathers from 72 breeding adults from six colonies (IDLE Fr and SJ; IM/FI GJ, RV, and SLI; and IP) with stable isotope analysis (see Table S3). Feathers are inert keratinous tissues and are similar in isotopic composition to the food that was taken up during molt in February–March. However, penguin molt is simultaneous and they fast during this period (Pütz et al., 2013), so feather stable isotope value composition reflects an integrated proxy of the geographic foraging areas used and the diet consumed during the pre‐molt foraging trip.

Feathers from each individual were cleaned using 2:1 chloroform–methanol solution, air‐dried, and then cut into small fragments. We wrapped ~0.6 mg of feather fragments into tin cups, which were combusted and analyzed for carbon and nitrogen isotopes (δ^13^C and δ^15^N) through a continuous flow stable isotope ratio mass spectrometer (CFIRMS) at Louisiana State University. *δ* notation is used and expressed in per mil units (‰), according to the following equation:

$$\delta X\;=\;[\left(R_{\text{sample}}/R_{\text{standard}}-1\right]\;\times \;1000.$$

where *X* is ^13^C or ^15^N and R is the corresponding ratio ^13^C/^12^C or ^15^N/^14^N. The R_standard_ values were based on Vienna Pee Dee Belemnite (VPDB) for δ^13^C and atmospheric N_2_ (AIR) for δ^15^N values. Raw δ values were normalized on a two‐point scale using glutamic acid reference materials with low and high values (i.e., USGS‐40: δ^13^C = −26.4‰, δ15N = −4.5‰; USGS‐41: δ^13^C = 37.6‰, δ^15^N = 47.6‰). Sample precision based on repeated reference materials was 0.1‰ both for δ^13^C and δ^15^N.

We used δ^13^C and δ^15^N values to calculate the Bayesian standard ellipse area (SEA_B_) as a measure of the isotopic foraging niche of each colony using the SIBER package (Jackson, Inger, Parnell, & Bearhop, 2011) in R software. SEA_B_ is the area calculated from iterative draws of ellipses from Markov chain Monte Carlo simulations, which outperforms other niche width estimations when sample sizes are unbalanced, albeit with greater uncertainty at small sample sizes (Jackson et al., 2011). Larger SEA_B_ values are interpreted as a wider isotopic foraging niche and denote the use of a wide range of foraging areas and/or prey resources. In contrast, smaller SEA_B_ values reflect a narrow isotopic foraging niche and the use of a narrow range of foraging areas and/or prey resources by a population. For each colony, we ran 10,000 iterations with default priors, discarding the first 1,000 as burn‐in. We then compared all ellipses of colonies in pairs yielding the proportion of SEA_B_ that are bigger than the other group's ellipses (PP). We consider PP > 0.95 to reflect relevant differences in SEA_B_ size.

To compare to what extent the isotopic foraging niche of each colony overlapped, we estimated the probability that individuals from one colony laid within the ellipses of another group using nicheROVER package (Swanson et al., 2015), producing values which range from 0 (no overlap) to 1 (complete overlap). Asymmetrical isotopic foraging niche overlap is defined as when one of the colonies presents a small isotopic niche which falls within a wider isotopic niche from another colony. We plotted both comparison with corrplot in R environment (Wei & Simko, 2017).

## 3 RESULTS

### 3.1 Genomic analyses

We recovered a total of 4,705 SNPs in 4,061 RAD loci across all individuals. We found evidence for two genetic clusters within the rockhopper, with the three sampled sites from IM/FI grouping with the northernmost, recently founded, continental colony, IP. The second genetic group comprised the three southernmost sampled colonies: Chi, IDLE‐BF, and IDLE‐SJ (Figure 1; Figure S1). We obtained the same result when using SNP or haplotype‐based analyses. In the hierarchical AMOVA that grouped samples by colonies, archipelagos, and northern/southern populations, only the highest level of clustering achieved statistical significance (Table 2). The average *F*_ST_ value between northern and southern clusters was 0.027 and consistently low among all SNPs (Figure S2). We did not find finer‐scale population structure when analysing colonies from each genetic cluster separately (Figures S3 and S4; Table S4).

**Table 2:** Hierarchical AMOVA grouped by genetic populations (northern and southern), archipelagos, and colonies.

	Df	Sum Sq	Mean Sq	Sigma	%	*p*
Between populations (North/ South)	1	1,501	1,501	11.96	2.57	**.001** *
Between archipelagos within pop	2	1,063	531	2.05	0.44	.084
Between colonies within archipelagos	3	1,380	460	−0.02	0.00	.292
Between samples within colonies	100	46,048	460	8.28	1.78	.115
Within samples	107	47,499	444	443.92	95.22	**.035** *
Total variations	213	97,491	458	466.19	100	

Note

* Indicates significant *p* < .05. Significant comparison highlighted in bold.

The demographic reconstruction found the effective population size of the southern group to be approximately two orders in magnitude larger than that of the northern group (Figure S5), consistent with the direction of the difference between census population sizes: ca. 500,000 pairs for the southern cluster and ca. 200,000 pairs for the northern one (Pütz et al., 2013). We also inferred an approximately fourfold decrease in effective population size with respect to the ancestral population. Although the 95% credible intervals for our estimates of effective population size were wide, the larger number inferred for the southern population was consistent with higher values of allelic diversity, heterozygosity, and lower kinship among individuals (Table 1; Figure S6). The northern and southern populations were inferred to have split in the order of hundreds to tens of thousands of generations, yet it is likely that the low differentiation between populations precluded a more precise estimate of their recent divergence time. Finally, we found higher migration in the north to south direction, although the credible intervals of this estimate were wide.

The individuals that were found dead on the coast of Santa Cruz (*n* = 21) and Tierra del Fuego (*n* = 7) were all assigned to the northern genetic cluster (Figure 1), indicating that the northern birds can be in proximity to the southern colonies where they forage but do not breed.

### 3.2 Stable isotope analysis

**Figure 2:**
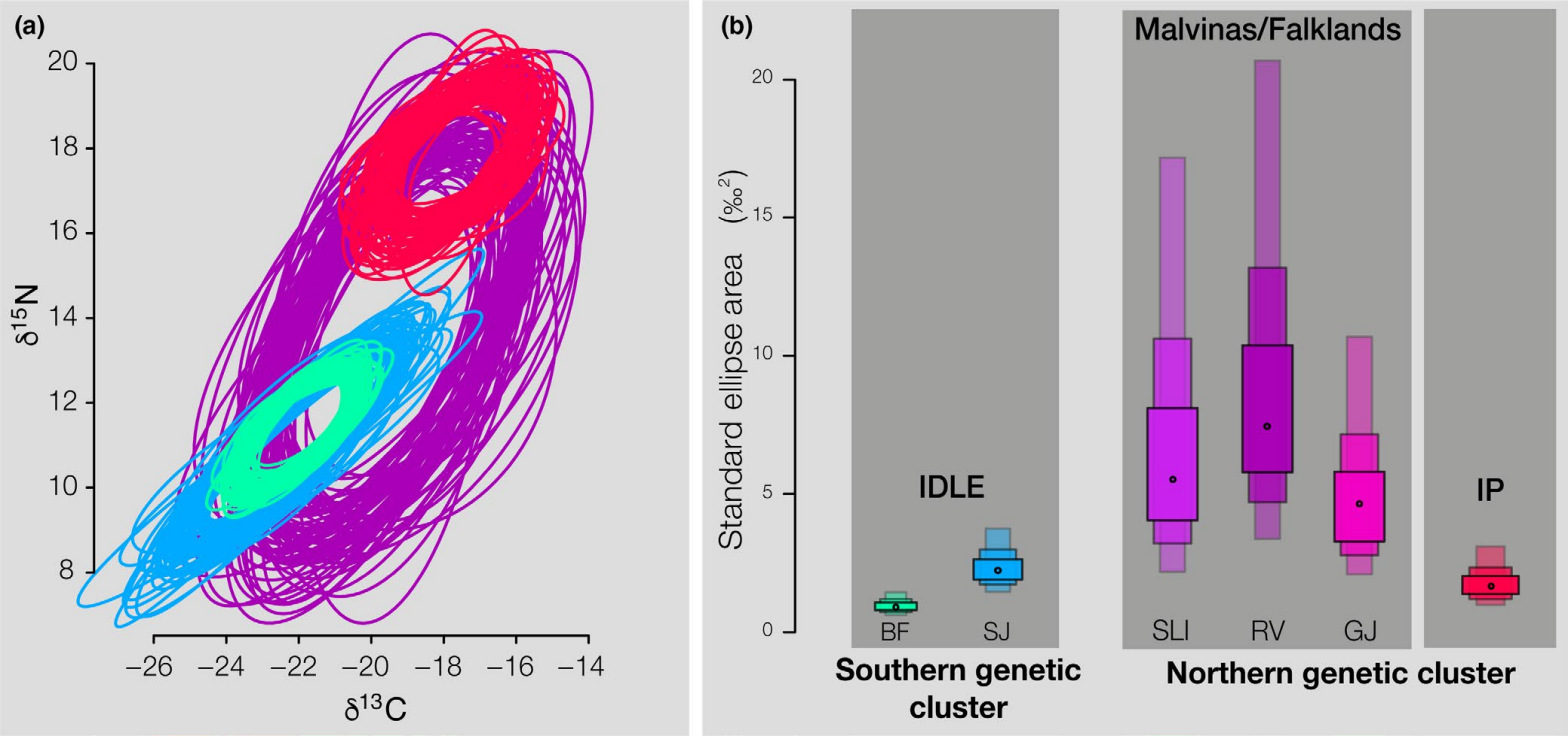
(a) Isotopic values for feathers from the 2016 pre‐molt period, ellipses show 100 SEA_B_ iterations for each archipelago (IM/FI—violet, IDLE‐SJ—cyan, IDLE‐BF—green, and IP—red), and (b) SEA_B_ size for the 6 colonies analyzed. Black dots represent mode, and boxes represent 50%, 75%, and 95% credible intervals from dark to light shades of each color. Colonies are color‐coded as in Figure 1

We found IM/FI colonies to have the largest isotopic foraging niche width, while IDLE‐BF represented the colony with the smallest niche width, and IDLE‐SJ and IP yielded intermediate values (Figure 2 and Figure S7). IM/FI colonies showed the widest isotopic niche, and they overlapped with all the other sites. Individuals from IDLE and IP were more likely to be contained in IM/FI ellipses than vice versa. IDLE showed high overlap within its colonies and no overlap with IP (Figure 2, Figure S8).

## 4 DISCUSSION

Philopatry is well‐documented and accepted as the main restriction for seabird species dispersal (Coulson, 2002). However, natal philopatry alone cannot account for the observed genetic pattern in the rockhopper, for which we found intraspecific genetic structure that grouped colonies into two clusters (northern and southern) in a highly vagile seabird, which opens intriguing questions on the processes that drive this recent isolation.

Previous studies have shown that nonphysical factors related to characteristics of the sea and breeding sites, as well as behavioral traits like travel distance and natal philopatry, can account for population structure in penguins (Clucas et al., 2018). Also, oceanographic fronts have been frequently related to population divergence in these species (e.g. Vianna et al., 2017). However, no evident frontal system exists (Acha, Mianzan, Guerrero, Favero, & Bava, 2004) between the two inferred clusters. Also, water masses are relatively homogeneous across the whole study area, encompassing mainly subantarctic shelf waters, that is southern Pacific and Antarctic Circumpolar Current water diluted by the freshwater input of the Chilean fjords and Magellan strait (Combes & Matano, 2014). In addition, given the swimming capabilities of this species (Pütz et al., 2013), currents are unlikely to drive dispersal direction. In fact, opposed to the south to north prevailing currents in the region (Palma, Matano, & Piola, 2008), we found higher migration in the opposite direction, although our estimates showed wide credible intervals probably due to low levels of differentiation (Figure S5c).

At‐sea range is also a potential factor explaining patterns of seabird intraspecific variation, with pelagic species showing less population structure than species with coastal habitats, where the probability of individuals of different populations coming into contact is lower (Clucas et al., 2018; Friesen, 2015). The rockhopper travels long distances during the breeding and nonbreeding season favoring population connectivity. However, foraging sites of different colonies during winter are nonoverlapping (Pütz et al., 2006), with IM/FI individuals using mainly the shelf and shelf break and IDLE birds foraging over the polar frontal zone. Both the segregation in winter foraging areas and the observed genetic pattern group individuals consistently, suggesting that individuals from colonies that share winter foraging sites are not genetically differentiated.

Our results also provide insights on the foraging niche of rockhoppers during the pre‐molt foraging trip, a key stage during which penguins replenish their reserves and prepare for the energetically demanding 2‐week whole body molt. We found differences in isotopic foraging niche across rockhopper colonies. When interpreting these results, it is important to understand that feather δ^13^C and δ^15^N values represent an integrated measure of both the geographic foraging areas used by penguin populations as well as the types of prey items they consume (Hinke et al., 2015). Within our study area, empirical (Lara, Alder, Franzosi, & Kattner, 2010) and modeling (Magozzi, Yool, Vander Zanden, Wunder, & Trueman, 2017) studies indicate a latitudinal trend in ecosystem δ^13^C and δ^15^N baselines (i.e., phytoplankton values), with lower δ^13^C and δ^15^N at higher latitudes. The rockhopper feather δ^13^C and δ^15^N values in our study matched this latitudinal trend, with northern colonies showing higher values than southern ones (Figure 2). Although we cannot fully disentangle the effects of forging area (i.e., latitude) and prey type (i.e., diet composition) on foraging niches, interesting aspects on the species foraging niche and connectivity become evident. Individuals of the recently founded IP colony have a much narrower isotopic niche than the source population IM/FI, albeit still included within the source population's isotopic niche. Plasticity in the use of foraging areas and prey types could allow dispersing birds to exploit a variety of resources and occupy new breeding areas as soon as they become available, which followed by local specialization could allow and enhance the foundation of new colonies.

Differences in foraging and migration behavior among colonial seabird species can also drive variations in key life traits like breeding behavior and phenology (Quillfeldt et al., 2019; Rayner et al., 2011), potentially driving population divergence (Friesen, 2015). Although rare among seabirds, differences in breeding phenology can generate isolation in numerous taxa, even in the absence of selective pressures (Hendry & Day, 2005). Differences in this life‐history trait between the northern (earlier) and southern cluster of ca. 15 days have been detected (Pütz et al., 2013), which could be limiting gene flow if there is a difference in the response to a phenological cue between individuals of the different clusters.

The mortality event we studied occurred in the Patagonian coast and was associated with unfavorable climatic conditions around IM/FI related to El Niño Southern Oscillation (Morgenthaler et al., 2018). Variable and/or uncertain conditions around a colony might promote higher dispersal rates, and “bad” years could increase the number of dispersing individuals. During “good” years, a lower prospecting behavior is expected but probably compensated by increased individual quality, which could also favor successful foundations. Thus, the occurrence of “bad” years could not necessarily be negative in terms of the species metapopulation dynamics and viability. Conditions that can trigger prospecting behavior in a certain colony may be related with high productivity, high intraspecific competition (i.e., high density), together with high environmental stochasticity. In seabird species in which colony foundations are so infrequent, this aspect should be explored further.

In a context of global human‐induced rapid environmental change (Sih, Ferrari, & Harris, 2011), behavioral plasticity plays a key role in population viability (Nussey, Kruuk, Morris, & Clutton‐Brock, 2007), especially in long‐lived species. Information on spatial foraging strategies, dispersal capabilities, and population structure give us certain predictability on environmental conditions that might threaten population viability. Given the low predictability of which environmental conditions could turn disadvantageous in the current global change scenario, an understanding of dispersal capabilities is critical to anticipate movement potential and range shifts of endangered species. Further studies are needed to analyze the prevalence of the pattern found in this study (i.e., colony‐specific foraging behavior and dispersal potential) in other sympatric seabird species, and on the past and current oceanographic conditions that could explain it.

## CONFLICT OF INTEREST

None declared.

## AUTHOR CONTRIBUTION

NAL, ARR, and BM conceived of the study, designed and coordinated the study, and drafted the manuscript. NAL, UB, KP, JAV, AM, EF, RSS, and ARR designed field work and collected data. NAL and LC carried out the molecular laboratory work, performed bioinformatic analyses, and drafted the manuscript. NAL, UB, and MJP performed isotopic analyses and drafted the manuscript. LC, UB, MJP, KP, JAV, ARR, and BM participated in data analysis and critically revised the manuscript. NAL wrote the paper. All authors revised the manuscript and gave final approval for publication.

### Open Research Badges

This article has been awarded <Open Materials, Open Data, Preregistered Research Designs> Badges. All materials and data are publicly accessible via the Open Science Framework at [https://doi.org/10.5061/dryad.vmcvdncpx; https://github.com/nlois/CodesMetapop].

## Data Availability

Stable isotope analysis raw data are available in Table S3 and genomic data are available at dryad repository (https://doi.org/10.5061/dryad.vmcvdncpx).
