# Supplementary material for: Metapopulation dynamics and foraging plasticity in a highly vagile seabird, the southern rockhopper penguin
Source: Ecol Evol. 2020 Mar 5;10(7):3346–55. doi: 10.1002/ece3.6127 (PMC7141044; doi:10.1002/ece3.6127)

Supplementary Figure 1. Population structure in the southern rockhopper penguin **(dataset: ALL)**
A. The principal component analysis based on 4975 SNPs with circles representing individuals and colours denoting colony of origin. The two outlier individuals separated by PC2 were found to be related with a kinship coefficient (r) of 0.5. These individuals were sampled as a breeding pair in IDLE-BF.
B. SVD quartets topology tree for ALL dataset. Related individuals con be denoted in both extremes of the tree
C. Structure plot for K=3, assigning individuals to either a Northern group (IP, IMF-GJ, IMF-RV, IMF-SLI), a Southern group (Chi, IDLE-BF, IDLE-SJ), or the previously mentioned related individuals from IDLE-BF.
D. Overall mean likelihood calculated from five runs ranging from K=1 to K=4.

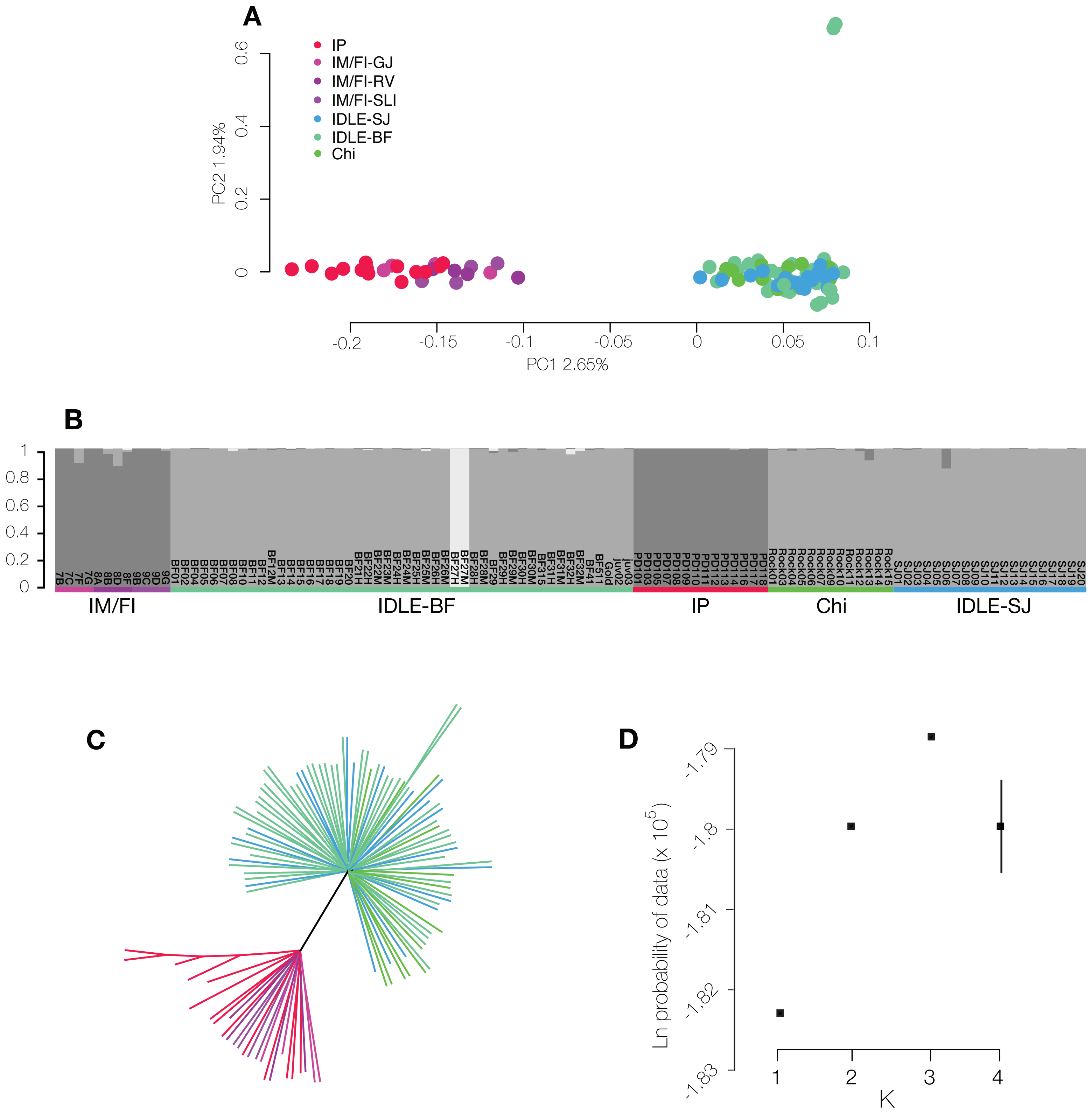

Supplementary Figure 1 (continued). Population structure in the southern rockhopper penguin **(dataset: ALL)** **E.** The fineRADstructure plot derived from haplotype data belonging to 4061 RAD loci indicates the presence of two genetic clusters.

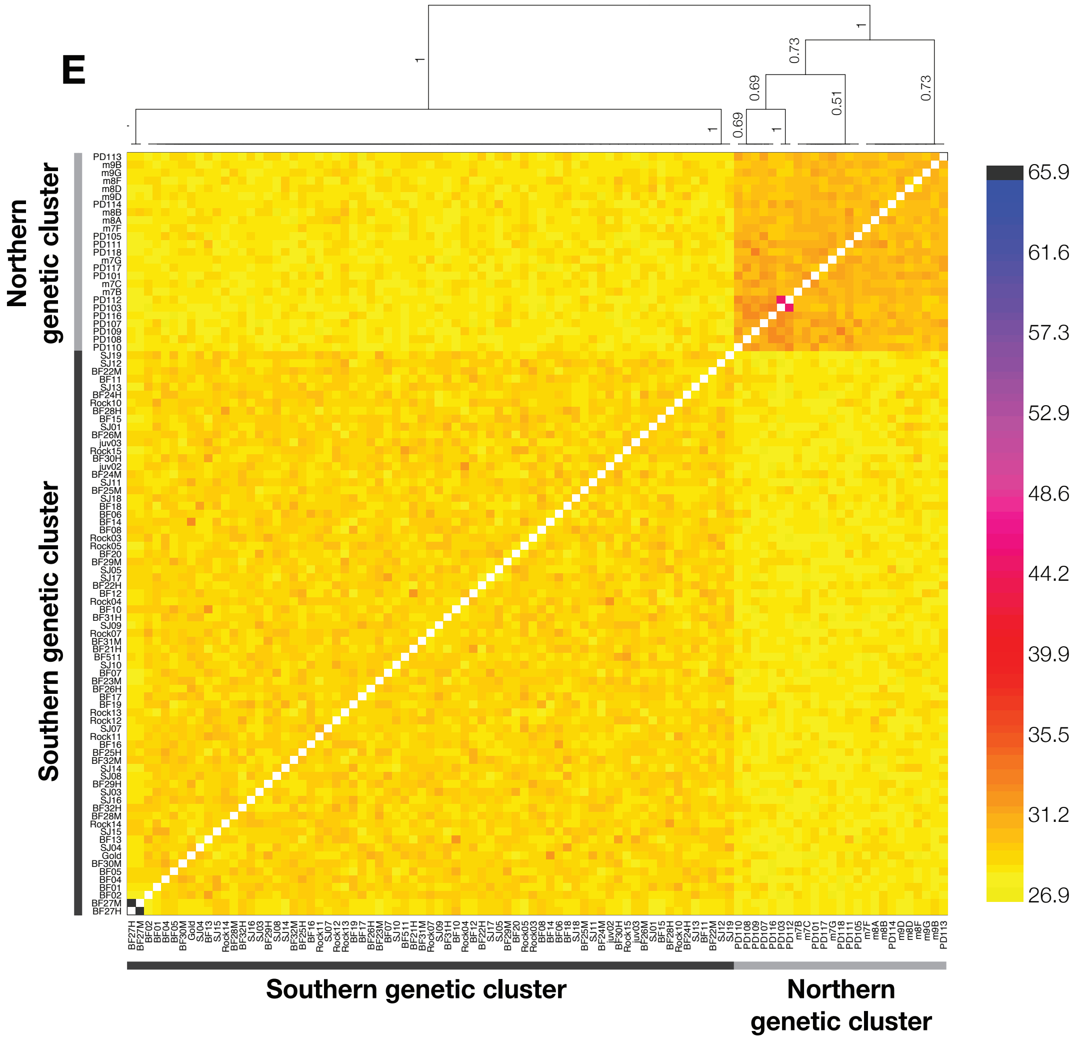

Supplementary Figure 2. Histogram showing the distribution of per-locus F_{ST} values between the Northern and Southern populations (4975 SNPs; average F_{ST} = 0.014 +/- 0.02, range = 0 – 0.27).

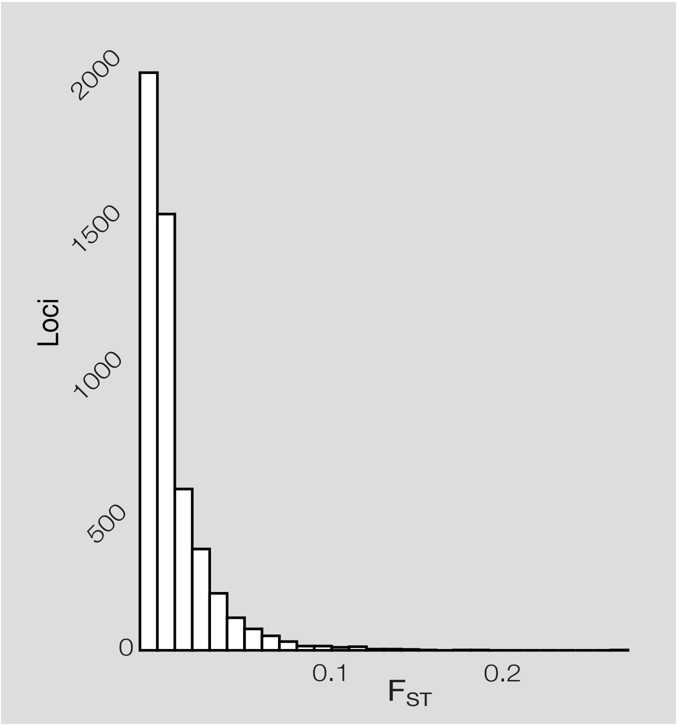

Supplementary Figure 3. Population genomic analysis within the Northern cluster **(dataset: NORTH):** PCA (**A**, 4572 SNPs), fineRADstructure analysis (**B**, 4228 RAD loci), Structure plot for K=2 (**C**, 2770 SNPs) with mean likelihood for each K (**D**) and SVDQuartets tree topology (**E**, 4572 SNPs) for 26 individuals from IP and IM/FI. All analyses cluster together two related males from IP with a kinship coefficient r = 0.26.

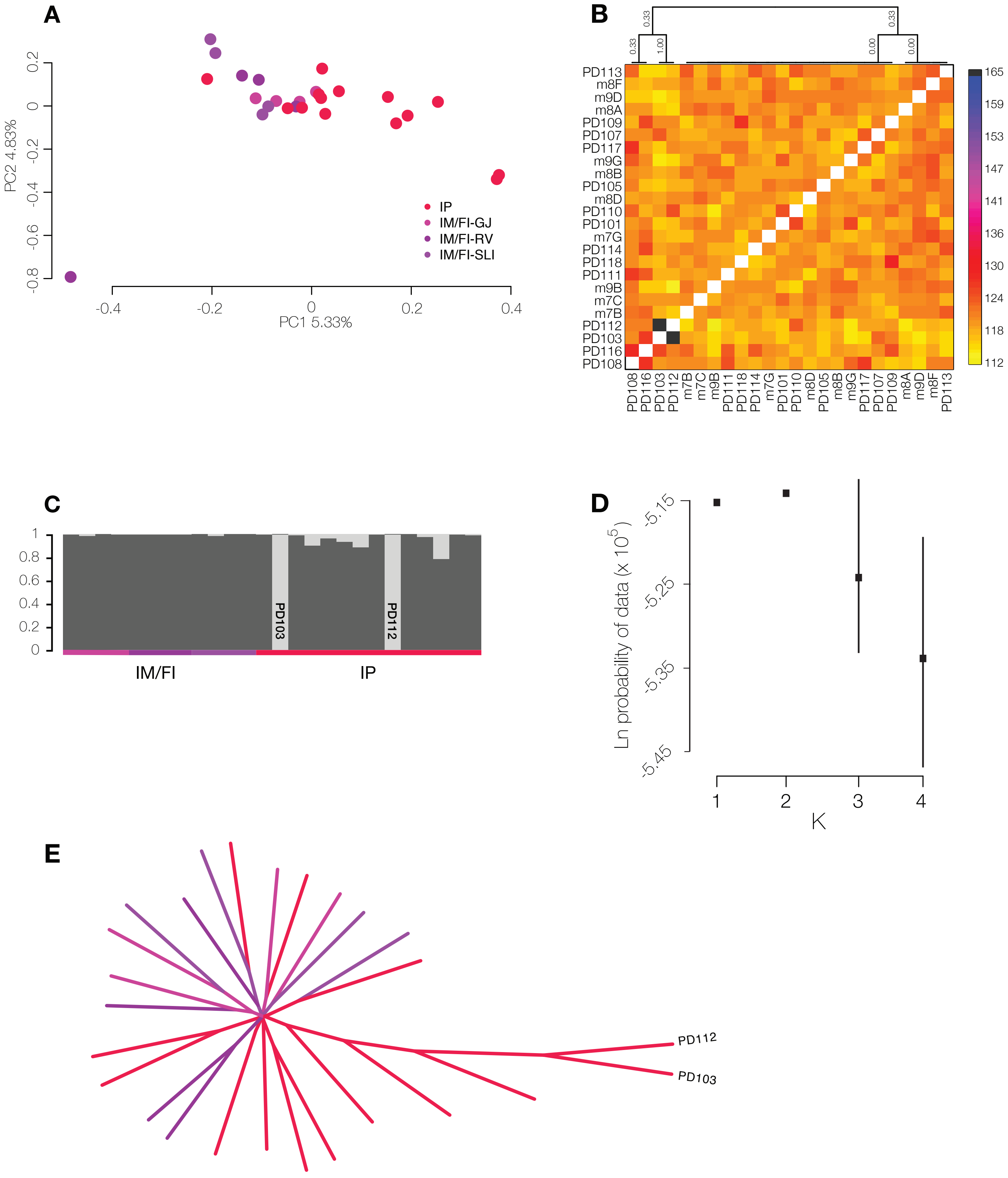

Supplementary Figure 4. Population genomic analysis within the Southern group **(dataset: SOUTH):** PCA (**A**, 4918 SNPs), fineRADstructure analysis (**B**, 3953 RAD loci), Structure plot for K=2 (**C**, 2718 SNPs) with mean likelihood (**D**) for each K, and tree topology (**E**, 4918 SNPs) for 81 individuals from IDLE and Chi. All analyses clustered two related individuals with a kinship coefficient r = 0.5 (sampled in IDLE-BF).

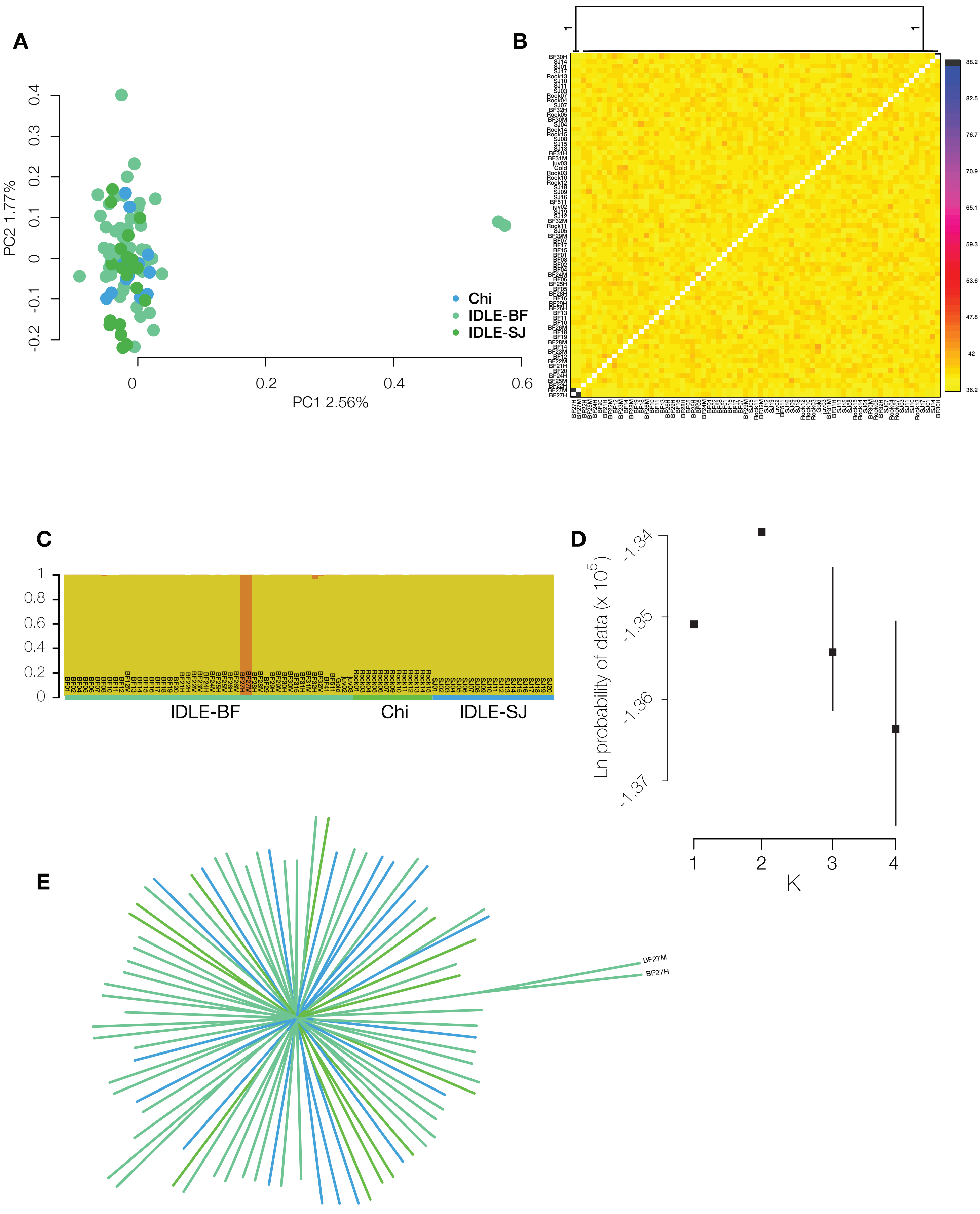

Supplementary Figure 5. G-PhoCS demographic modelling. Co-estimation of current and ancestral population sizes (**A**), splitting time between the Northern and Southern groups (**B**), and bi-directional migration (**C**).

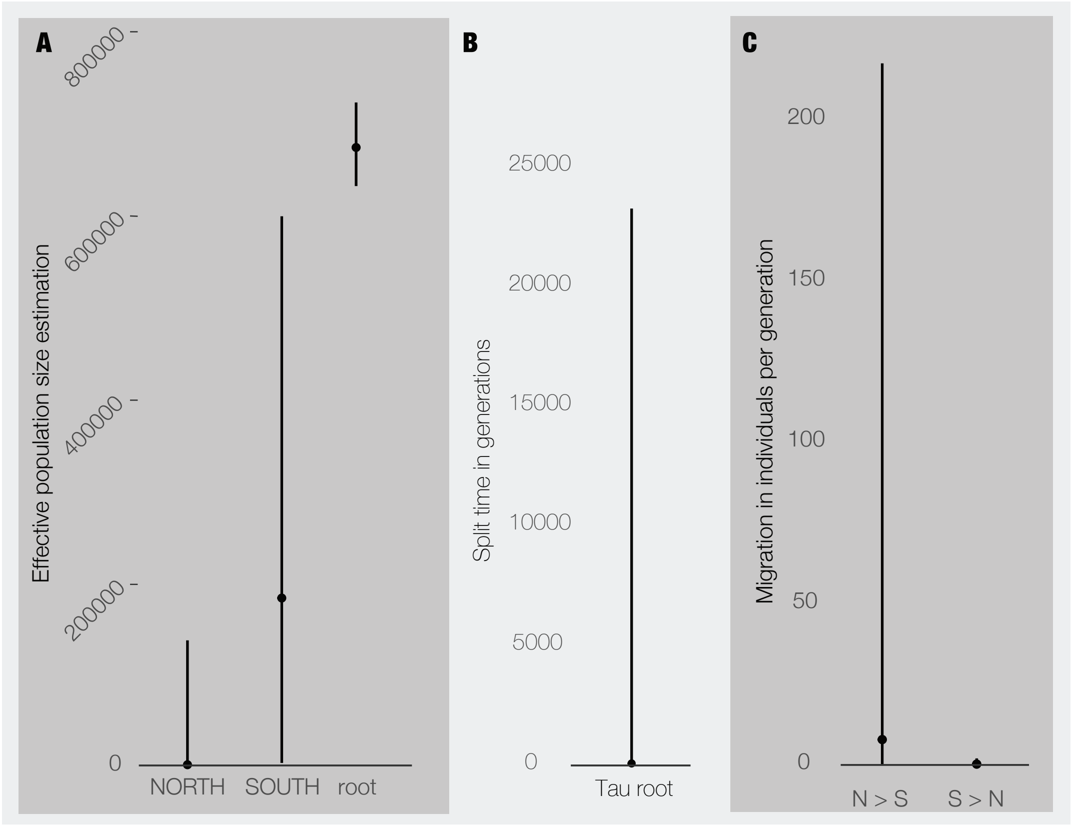

Supplementary Figure 6. Distribution of pairwise values of estimated kinship (r) within and between individuals from the Northern and Southern genetic populations.

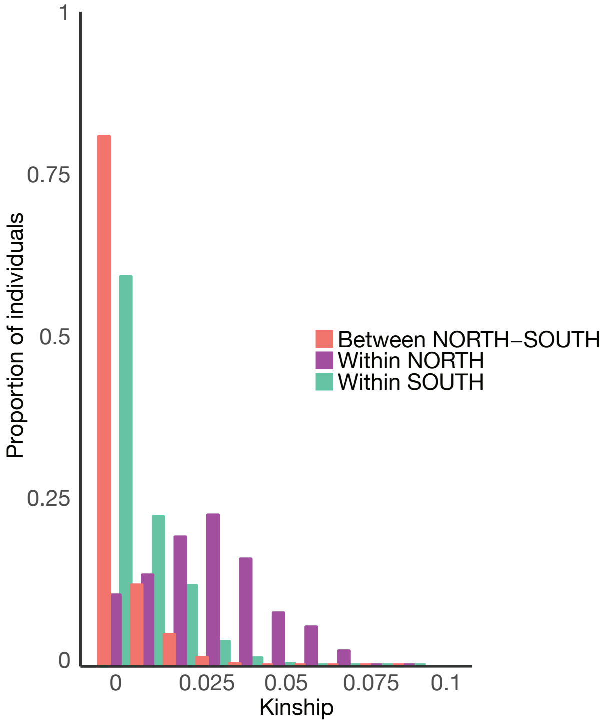

Supplementary Figure 7. Probability of isotopic niche area of colony in column is bigger than the colony in the row for each model.

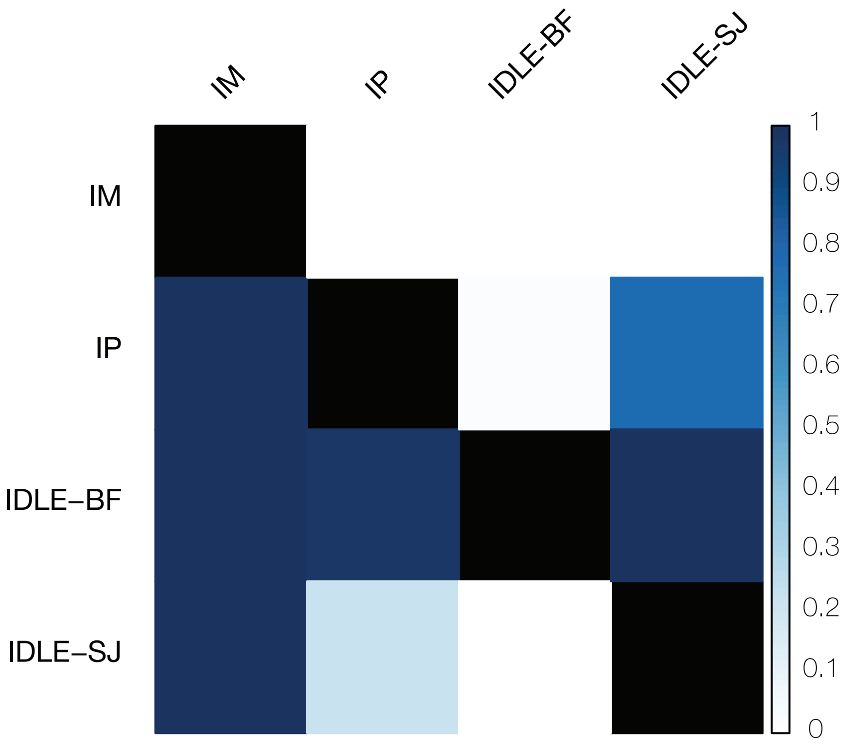

Supplementary Figure 8. Isotopic niche overlap between groups estimated as the probability of each individual from the colony in the column to be contained within the isotopic niche of the colony in the row. Mean overlap is represented by colour, and confidence interval presented within each cell.

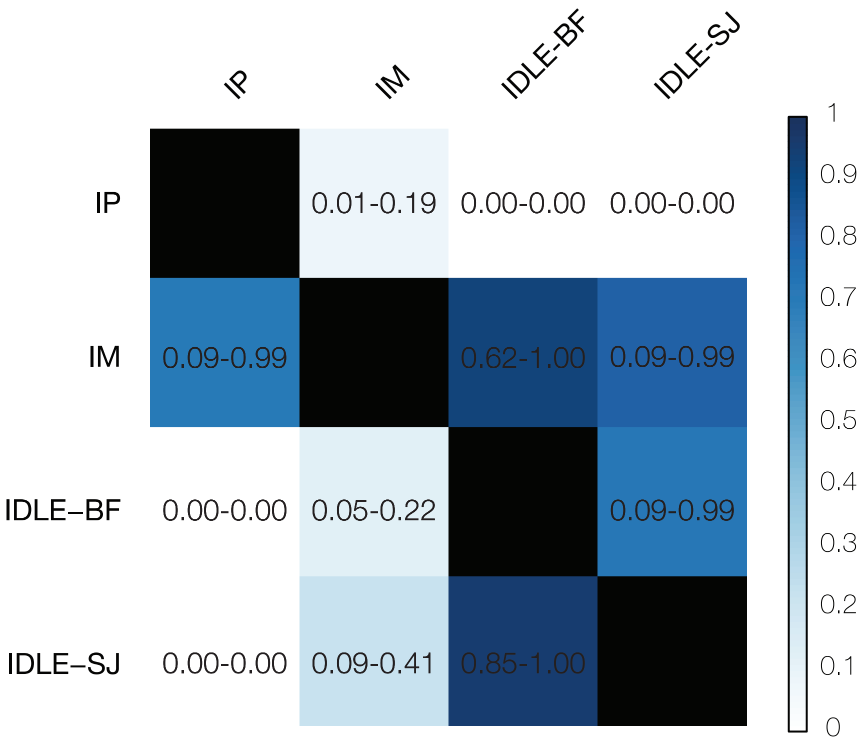

Supplement: Supplementary file 1 [file ECE3-10-3346-s001.pdf]
