# Supplementary material for: Metapopulation dynamics and foraging plasticity in a highly vagile seabird, the southern rockhopper penguin
Source: Ecol Evol. 2020 Mar 5;10(7):3346–55. doi: 10.1002/ece3.6127 (PMC7141044; doi:10.1002/ece3.6127)
Supplement: Supplementary file 2 [file ECE3-10-3346-s002.pdf]

Supplementary Table 1. Details for all individuals sampled and genotyped.

	Code	Tissue	Age	Site	Archipelago	Lat	Long	DATASET				% missing data
								ALL	ALL+ DEAD	NORTH	SOUTH	
1	PD108	Blood	Adult	Isla Pingüino	Isla Pingüino	47°55' S	65°43' W	X	X	X		2.83
2	PD116	Blood	Adult	Isla Pingüino	Isla Pingüino	47°55' S	65°43' W	X	X	X		2.70
3	PD114	Blood	Adult	Isla Pingüino	Isla Pingüino	47°55' S	65°43' W	X	X	X		1.13
4	PD101	Blood	Adult	Isla Pingüino	Isla Pingüino	47°55' S	65°43' W	X	X	X		1.01
5	PD109	Blood	Adult	Isla Pingüino	Isla Pingüino	47°55' S	65°43' W	X	X	X		0.88
6	PD112	Blood	Adult	Isla Pingüino	Isla Pingüino	47°55' S	65°43' W	X	X	X		0.82
7	PD113	Blood	Adult	Isla Pingüino	Isla Pingüino	47°55' S	65°43' W	X	X	X		0.50
8	PD110	Blood	Adult	Isla Pingüino	Isla Pingüino	47°55' S	65°43' W	X	X	X		0.44
9	PD107	Blood	Adult	Isla Pingüino	Isla Pingüino	47°55' S	65°43' W	X	X	X		0.38
10	PD111	Blood	Adult	Isla Pingüino	Isla Pingüino	47°55' S	65°43' W	X	X	X		0.38
11	PD117	Blood	Adult	Isla Pingüino	Isla Pingüino	47°55' S	65°43' W	X	X	X		0.38
12	PD118	Blood	Adult	Isla Pingüino	Isla Pingüino	47°55' S	65°43' W	X	X	X		0.38
13	PD105	Blood	Adult	Isla Pingüino	Isla Pingüino	47°55' S	65°43' W	X	X	X		0.31
14	PD103	Blood	Adult	Isla Pingüino	Isla Pingüino	47°55' S	65°43' W	X	X	X		0.19
15	7F	Blood	Adult	Grand Jason	Malvinas/Falklands	51°03' S	61°06' W	X	X	X		11.75
16	7C	Blood	Adult	Grand Jason	Malvinas/Falklands	51°03' S	61°06' W	X	X	X		6.98
17	7G	Blood	Adult	Grand Jason	Malvinas/Falklands	51°03' S	61°06' W	X	X	X		1.13
18	7B	Blood	Adult	Grand Jason	Malvinas/Falklands	51°03' S	61°06' W	X	X	X		0.50
19	9C	Blood	Adult	Rookery Valley	Malvinas/Falklands	51°36' S	57°54' W	X	X	X		17.41
20	9D	Blood	Adult	Rookery Valley	Malvinas/Falklands	51°36' S	57°54' W	X	X	X		2.20
21	9G	Blood	Adult	Rookery Valley	Malvinas/Falklands	51°36' S	57°54' W	X	X	X		0.69
22	9B	Blood	Adult	Rookery Valley	Malvinas/Falklands	51°36' S	57°54' W	X	X	X		0.63
23	8F	Blood	Adult	Sea Lion Island	Malvinas/Falklands	52°26' S	59°07' W	X	X	X		5.66
24	8D	Blood	Adult	Sea Lion Island	Malvinas/Falklands	52°26' S	59°07' W	X	X	X		1.01
25	8B	Blood	Adult	Sea Lion Island	Malvinas/Falklands	52°26' S	59°07' W	X	X	X		0.69
26	8A	Blood	Adult	Sea Lion Island	Malvinas/Falklands	52°26' S	59°07' W	X	X	X		0.50
27	BF315	Blood	Adult	Bahía Franklin	De los Estados	54°52' S	64°41' W	X	X		X	43.37
28	BF29	Blood	Adult	Bahía Franklin	De los Estados	54°52' S	64°41' W	X	X		X	33.31
29	BF12M	Blood	Adult	Bahía Franklin	De los Estados	54°52' S	64°41' W	X	X		X	20.49
30	BF41	Blood	Adult	Bahía Franklin	De los Estados	54°52' S	64°41' W	X	X		X	12.95
31	BF07	Blood	Adult	Bahía Franklin	De los Estados	54°52' S	64°41' W	X	X		X	7.98
32	BF24H	Blood	Adult	Bahía Franklin	De los Estados	54°52' S	64°41' W	X	X		X	6.85

	Code	Tissue	Age	Site	Archipelago	Lat	Long	DATASET ALL	ALL+ DEAD	NORTH	SOUTH	% missing data
33	BF511	Blood	Adult	Bahía Franklin	De los Estados	54°52' S	64°41' W	X	X		X	3.71
34	BF24M	Blood	Adult	Bahía Franklin	De los Estados	54°52' S	64°41' W	X	X		X	3.02
35	BF28H	Blood	Adult	Bahía Franklin	De los Estados	54°52' S	64°41' W	X	X		X	3.02
36	BF17	Blood	Adult	Bahía Franklin	De los Estados	54°52' S	64°41' W	X	X		X	2.64
37	BF04	Blood	Adult	Bahía Franklin	De los Estados	54°52' S	64°41' W	X	X		X	2.33
38	BF12	Blood	Adult	Bahía Franklin	De los Estados	54°52' S	64°41' W	X	X		X	2.20
39	BF26H	Blood	Adult	Bahía Franklin	De los Estados	54°52' S	64°41' W	X	X		X	1.76
40	BF22H	Blood	Adult	Bahía Franklin	De los Estados	54°52' S	64°41' W	X	X		X	1.51
41	BF_Gold	Blood	Adult	Bahía Franklin	De los Estados	54°52' S	64°41' W	X	X		X	1.26
42	BF32H	Blood	Adult	Bahía Franklin	De los Estados	54°52' S	64°41' W	X	X		X	1.13
43	BF01	Blood	Adult	Bahía Franklin	De los Estados	54°52' S	64°41' W	X	X		X	1.07
44	BF26M	Blood	Adult	Bahía Franklin	De los Estados	54°52' S	64°41' W	X	X		X	1.07
45	BF29H	Blood	Adult	Bahía Franklin	De los Estados	54°52' S	64°41' W	X	X		X	1.07
46	BF06	Blood	Adult	Bahía Franklin	De los Estados	54°52' S	64°41' W	X	X		X	0.88
47	BF08	Blood	Adult	Bahía Franklin	De los Estados	54°52' S	64°41' W	X	X		X	0.88
48	BF27H	Blood	Adult	Bahía Franklin	De los Estados	54°52' S	64°41' W	X	X		X	0.88
49	BF05	Blood	Adult	Bahía Franklin	De los Estados	54°52' S	64°41' W	X	X		X	0.82
50	BF25H	Blood	Adult	Bahía Franklin	De los Estados	54°52' S	64°41' W	X	X		X	0.82
51	BF02	Blood	Adult	Bahía Franklin	De los Estados	54°52' S	64°41' W	X	X		X	0.75
52	BF13	Blood	Adult	Bahía Franklin	De los Estados	54°52' S	64°41' W	X	X		X	0.75
53	BF18	Blood	Adult	Bahía Franklin	De los Estados	54°52' S	64°41' W	X	X		X	0.75
54	BF31H	Blood	Adult	Bahía Franklin	De los Estados	54°52' S	64°41' W	X	X		X	0.75
55	BF11	Blood	Adult	Bahía Franklin	De los Estados	54°52' S	64°41' W	X	X		X	0.69
56	BF16	Blood	Adult	Bahía Franklin	De los Estados	54°52' S	64°41' W	X	X		X	0.69
57	BF22M	Blood	Adult	Bahía Franklin	De los Estados	54°52' S	64°41' W	X	X		X	0.69
58	BF30M	Blood	Adult	Bahía Franklin	De los Estados	54°52' S	64°41' W	X	X		X	0.63
59	BF10	Blood	Adult	Bahía Franklin	De los Estados	54°52' S	64°41' W	X	X		X	0.57
60	BF14	Blood	Adult	Bahía Franklin	De los Estados	54°52' S	64°41' W	X	X		X	0.57
61	BF19	Blood	Adult	Bahía Franklin	De los Estados	54°52' S	64°41' W	X	X		X	0.57
62	BF23M	Blood	Adult	Bahía Franklin	De los Estados	54°52' S	64°41' W	X	X		X	0.50
63	BF28M	Blood	Adult	Bahía Franklin	De los Estados	54°52' S	64°41' W	X	X		X	0.50
64	BF31M	Blood	Adult	Bahía Franklin	De los Estados	54°52' S	64°41' W	X	X		X	0.50

	Code	Tissue	Age	Site	Archipelago	Lat	Long	DATASET				% missing data
								ALL	ALL+ DEAD	NORTH	SOUTH	
65	BF15	Blood	Adult	Bahía Franklin	De los Estados	54°52' S	64°41' W	X	X		X	0.44
66	BF21H	Blood	Adult	Bahía Franklin	De los Estados	54°52' S	64°41' W	X	X		X	0.44
67	BF25M	Blood	Adult	Bahía Franklin	De los Estados	54°52' S	64°41' W	X	X		X	0.44
68	BF20	Blood	Adult	Bahía Franklin	De los Estados	54°52' S	64°41' W	X	X		X	0.38
69	BF27M	Blood	Adult	Bahía Franklin	De los Estados	54°52' S	64°41' W	X	X		X	0.38
70	BF30H	Blood	Adult	Bahía Franklin	De los Estados	54°52' S	64°41' W	X	X		X	0.38
71	BF29M	Blood	Adult	Bahía Franklin	De los Estados	54°52' S	64°41' W	X	X		X	0.31
72	BF32M	Blood	Adult	Bahía Franklin	De los Estados	54°52' S	64°41' W	X	X		X	0.25
73	juv03	Blood	Juvenile	Bahía Franklin	De los Estados	54°52' S	64°41' W	X	X		X	0.88
74	juv02	Blood	Juvenile	Bahía Franklin	De los Estados	54°52' S	64°41' W	X	X		X	0.57
75	SJ06	Blood	Adult	San Juan	De los Estados	54°44' S	63°50' W	X	X		X	47.83
76	SJ20	Blood	Adult	San Juan	De los Estados	54°44' S	63°50' W	X	X		X	41.80
77	SJ02	Blood	Adult	San Juan	De los Estados	54°44' S	63°50' W	X	X		X	33.88
78	SJ11	Blood	Adult	San Juan	De los Estados	54°44' S	63°50' W	X	X		X	4.34
79	SJ08	Blood	Adult	San Juan	De los Estados	54°44' S	63°50' W	X	X		X	2.39
80	SJ04	Blood	Adult	San Juan	De los Estados	54°44' S	63°50' W	X	X		X	2.14
81	SJ12	Blood	Adult	San Juan	De los Estados	54°44' S	63°50' W	X	X		X	2.14
82	SJ09	Blood	Adult	San Juan	De los Estados	54°44' S	63°50' W	X	X		X	1.76
83	SJ15	Blood	Adult	San Juan	De los Estados	54°44' S	63°50' W	X	X		X	1.19
84	SJ16	Blood	Adult	San Juan	De los Estados	54°44' S	63°50' W	X	X		X	1.07
85	SJ10	Blood	Adult	San Juan	De los Estados	54°44' S	63°50' W	X	X		X	0.88
86	SJ18	Blood	Adult	San Juan	De los Estados	54°44' S	63°50' W	X	X		X	0.82
87	SJ19	Blood	Adult	San Juan	De los Estados	54°44' S	63°50' W	X	X		X	0.82
88	SJ03	Blood	Adult	San Juan	De los Estados	54°44' S	63°50' W	X	X		X	0.75
89	SJ14	Blood	Adult	San Juan	De los Estados	54°44' S	63°50' W	X	X		X	0.75
90	SJ07	Blood	Adult	San Juan	De los Estados	54°44' S	63°50' W	X	X		X	0.69
91	SJ13	Blood	Adult	San Juan	De los Estados	54°44' S	63°50' W	X	X		X	0.69
92	SJ17	Blood	Adult	San Juan	De los Estados	54°44' S	63°50' W	X	X		X	0.69
93	SJ01	Blood	Adult	San Juan	De los Estados	54°44' S	63°50' W	X	X		X	0.50
94	SJ05	Blood	Adult	San Juan	De los Estados	54°44' S	63°50' W	X	X		X	0.44
95	Rock06	Blood	Adult	Isla Terhalten	Southern Chile	55°27' S	67°03' W	X	X		X	44.00
96	Rock01	Blood	Adult	Isla Terhalten	Southern Chile	55°27' S	67°03' W	X	X		X	38.40

	Code	Tissue	Age	Site	Archipelago	Lat	Long	DATASET<br>ALL	ALL+ DEAD	NORTH	SOUTH	% missing data
97	Rock09	Blood	Adult	Isla Terhalten	Southern Chile	55°27' S	67°03' W	X	X		X	18.04
98	Rock04	Blood	Adult	Isla Terhalten	Southern Chile	55°27' S	67°03' W	X	X		X	11.94
99	Rock05	Blood	Adult	Isla Terhalten	Southern Chile	55°27' S	67°03' W	X	X		X	9.24
100	Rock10	Blood	Adult	Isla Terhalten	Southern Chile	55°27' S	67°03' W	X	X		X	8.67
101	Rock13	Blood	Adult	Isla Terhalten	Southern Chile	55°27' S	67°03' W	X	X		X	7.10
102	Rock03	Blood	Adult	Isla Terhalten	Southern Chile	55°27' S	67°03' W	X	X		X	3.39
103	Rock07	Blood	Adult	Isla Terhalten	Southern Chile	55°27' S	67°03' W	X	X		X	1.89
104	Rock14	Blood	Adult	Isla Terhalten	Southern Chile	55°27' S	67°03' W	X	X		X	1.32
105	Rock12	Blood	Adult	Isla Terhalten	Southern Chile	55°27' S	67°03' W	X	X		X	1.19
106	Rock11	Blood	Adult	Isla Terhalten	Southern Chile	55°27' S	67°03' W	X	X		X	1.13
107	Rock15	Blood	Adult	Isla Terhalten	Southern Chile	55°27' S	67°03' W	X	X		X	0.75
108	MD07	Skin	Dead Adult	SC coast	Unknown				X			25.02
109	MD05	Skin	Dead Adult	SC coast	Unknown				X			11.00
110	MDB	Muscle	Dead Adult	SC coast	Unknown				X			6.73
111	MDIP	Muscle	Dead Adult	SC coast	Unknown				X			5.91
112	MD03	Skin	Dead Adult	SC coast	Unknown				X			4.53
113	MDC	Muscle	Dead Adult	SC coast	Unknown				X			4.21
114	MDJ	Muscle	Dead Adult	SC coast	Unknown				X			3.96
115	MDM	Muscle	Dead Adult	SC coast	Unknown				X			3.65
116	MDO	Muscle	Dead Adult	SC coast	Unknown				X			3.33
117	MD04	Skin	Dead Adult	SC coast	Unknown				X			2.39
118	MD13	Muscle	Dead Adult	SC coast	Unknown				X			1.95
119	MD11	Muscle	Dead Adult	SC coast	Unknown				X			1.63
120	MDL	Muscle	Dead Adult	SC coast	Unknown				X			1.45
121	MD01	Skin	Dead Adult	SC coast	Unknown				X			1.07
122	MD14	Muscle	Dead Adult	SC coast	Unknown				X			1.07
123	MDP	Muscle	Dead Adult	SC coast	Unknown				X			1.01
124	MD12	Muscle	Dead Adult	SC coast	Unknown				X			0.88
125	MDA	Muscle	Dead Adult	SC coast	Unknown				X			0.75
126	MD02	Skin	Dead Adult	SC coast	Unknown				X			0.63
127	MDN	Muscle	Dead Adult	SC coast	Unknown				X			0.63
128	MDH	Muscle	Dead Adult	SC coast	Unknown				X			0.44

	Code	Tissue	Age	Site	Archipelago	Lat	Long	DATASET				% missing data
								ALL	ALL+ DEAD	NORTH	SOUTH	
129	M07	Muscle	Dead Adult	TdF coast	Unknown				**X**			34.70
130	MDK	Muscle	Dead Adult	TdF coast	Unknown				**X**			24.58
131	M04	Muscle	Dead Adult	TdF coast	Unknown				**X**			22.06
132	M09	Muscle	Dead Adult	TdF coast	Unknown				**X**			14.52
133	M08	Muscle	Dead Adult	TdF coast	Unknown				**X**			4.46
134	M02	Muscle	Dead Adult	TdF coast	Unknown				**X**			1.95
135	M10	Muscle	Dead Adult	TdF coast	Unknown				**X**			1.01
136	BF03	Blood	Adult	Bahía Franklin	De los Estados	54°52' S	64°41' W	**Excluded**				99.94
137	BF23H	Blood	Adult	Bahía Franklin	De los Estados	54°52' S	64°41' W	**Excluded**				92.02
138	BF13M	Blood	Adult	Bahía Franklin	De los Estados	54°52' S	64°41' W	**Excluded**				85.36
139	BF21M	Blood	Adult	Bahía Franklin	De los Estados	54°52' S	64°41' W	**Excluded**				64.30
140	BF26CH	Blood	Chick	Bahía Franklin	De los Estados	54°52' S	64°41' W	**Excluded**				99.06
141	BF27CH	Blood	Chick	Bahía Franklin	De los Estados	54°52' S	64°41' W	**Excluded**				55.19
142	BF24CH	Blood	Chick	Bahía Franklin	De los Estados	54°52' S	64°41' W	**Excluded**				43.31
143	29Ch	Blood	Chick	Bahía Franklin	De los Estados	54°52' S	64°41' W	**Excluded**				37.02
144	BF23CH	Blood	Chick	Bahía Franklin	De los Estados	54°52' S	64°41' W	**Excluded**				0.38
145	BF30CH	Blood	Chick	Bahía Franklin	De los Estados	54°52' S	64°41' W	**Excluded**				0.38
146	BF31CH	Blood	Chick	Bahía Franklin	De los Estados	54°52' S	64°41' W	**Excluded**				0.38
147	BF32CH	Blood	Chick	Bahía Franklin	De los Estados	54°52' S	64°41' W	**Excluded**				0.38
148	juv01	Blood	Juvenile	Bahía Franklin	De los Estados	54°52' S	64°41' W	**Excluded**				99.06
149	juv04	Blood	Juvenile	Bahía Franklin	De los Estados	54°52' S	64°41' W	**Excluded**				51.29
150	PD119	Blood	Chick	Isla Pingüino	Isla Pingüino	47°55' S	65°43' W	**Excluded**				3.14
151	PD102	Blood	Chick	Isla Pingüino	Isla Pingüino	47°55' S	65°43' W	**Excluded**				0.69
152	PD115	Blood	Chick	Isla Pingüino	Isla Pingüino	47°55' S	65°43' W	**Excluded**				0.69
153	Rock02	Blood	Adult	Isla Terhalten	Southern Chile	54°52' S	64°41' W	**Excluded**				60.21
154	Rock08	Blood	Adult	Isla Terhalten	Southern Chile	54°52' S	64°41' W	**Excluded**				57.32
155	M01	Muscle	Dead Adult	TdF coast	Unknown			**Excluded**				99.94
156	M05	Muscle	Dead Adult	TdF coast	Unknown			**Excluded**				99.94
157	M03	Muscle	Dead Adult	TdF coast	Unknown			**Excluded**				96.92
158	MD06	Skin	Dead Adult	SC coast	Unknown			**Excluded**				88.18
159	M06	Skin	Dead Adult	TdF coast	Unknown			**Excluded**				NA
160	M11	Skin	Dead Adult	TdF coast	Unknown			**Excluded**				NA

Supplementary Table 2. Datasets used, sample sizes and analyses carried out throughout the population genomic analyses. *1 Carried out only for ALL, NORTH and SOUTH. *2 Carried out for ALL dataset with one haplotype per individual and 85 individuals (26 NORTH and 59 SOUTH randomly selected).

Dataset	Colonies	n	All SNPs	SNPs (1 per locus)	loci
ALL	All	107	4975	2779	4061
NORTH	IM/FI + IP	26	4572	2770	4228
SOUTH	IDLE + Chi	81	4918	2718	3953
ALLandDead	All + stranded individual	135	4705	2645	3882
**Analyses**			PCA, AMOVA*1	STRUCTURE, fineRADstructure, GPhoCS*2	

Supplementary Table 3. Details for the individuals sampled and analysed for isotopic composition. δ13C, δ15N and C/N ratio is reported.

**Code**	**Archipelago**	**Colony**	**δ_{13}C**	**δ_{15}N**	**C/N**
PD118H	Continent	Isla Pinguino	-17.31	17.88	3.25
PD117M	Continent	Isla Pinguino	-18.10	17.62	3.29
PD114M	Continent	Isla Pinguino	-15.91	18.21	3.15
PD113H	Continent	Isla Pinguino	-17.77	18.34	3.22
PD112M	Continent	Isla Pinguino	-18.89	17.03	3.19
PD111H	Continent	Isla Pinguino	-17.56	17.76	3.34
PD110H	Continent	Isla Pinguino	-17.74	17.78	3.17
PD109M	Continent	Isla Pinguino	-17.76	16.97	3.18
PD108H	Continent	Isla Pinguino	-17.87	17.40	3.34
PD107M	Continent	Isla Pinguino	-19.16	18.22	3.25
PD105M	Continent	Isla Pinguino	-16.94	18.92	3.16
PD103M	Continent	Isla Pinguino	-17.38	18.33	3.32
PD116H	Continent	Isla Pinguino	-18.87	15.95	3.34
BF27H	IDLE	Bahia Franklin	-22.65	10.68	3.15
BF27M	IDLE	Bahia Franklin	-20.81	12.83	3.14
BF28H	IDLE	Bahia Franklin	-21.75	11.36	3.22
BF29M	IDLE	Bahia Franklin	-22.52	10.88	3.16
BF29H	IDLE	Bahia Franklin	-20.99	11.92	3.15
BF5	IDLE	Bahia Franklin	-22.32	11.80	3.20
BF11	IDLE	Bahia Franklin	-20.63	12.21	3.10
BF13	IDLE	Bahia Franklin	-22.26	10.77	3.12
BF20	IDLE	Bahia Franklin	-21.80	11.39	3.20
BFcam21M	IDLE	Bahia Franklin	-21.57	12.03	3.18
BFcam21H	IDLE	Bahia Franklin	-22.88	11.33	3.13
BFcam22H	IDLE	Bahia Franklin	-22.95	10.55	3.12
BFcam23H	IDLE	Bahia Franklin	-21.69	11.56	3.20
BFcam25H	IDLE	Bahia Franklin	-21.71	11.80	3.12
BFcam26M	IDLE	Bahia Franklin	-21.70	10.97	3.17
BFcam23M	IDLE	Bahia Franklin	-21.41	11.17	3.17
BFcam25M	IDLE	Bahia Franklin	-22.66	11.00	3.41
BFcam26H	IDLE	Bahia Franklin	-21.86	11.63	3.16
BF18	IDLE	Bahia Franklin	-23.07	10.56	3.25
BFcam22M	IDLE	Bahia Franklin	-22.32	10.69	3.17
BFcam28M	IDLE	Bahia Franklin	-23.16	10.94	3.52
BF1	IDLE	Bahia Franklin	-22.79	11.46	3.21
SJ3	IDLE	Bahia Franklin	-21.15	12.37	3.25

SJ2	IDLE	San Juan	-23.37	10.39	3.29
SJ3	IDLE	San Juan	-22.41	10.85	3.20
SJ4	IDLE	San Juan	-23.24	10.12	3.37
SJ5	IDLE	San Juan	-22.89	9.96	3.18
SJ6	IDLE	San Juan	-17.92	14.41	3.27
SJ7	IDLE	San Juan	-20.71	12.42	3.29
SJ8	IDLE	San Juan	-22.85	10.51	3.19
SJ10	IDLE	San Juan	-22.94	10.83	3.18
SJ11	IDLE	San Juan	-23.12	11.29	3.20
SJ12	IDLE	San Juan	-23.23	10.59	3.40
SJ14	IDLE	San Juan	-21.90	10.54	3.16
SJ15	IDLE	San Juan	-22.63	10.40	3.19
SJ16	IDLE	San Juan	-22.99	11.15	3.23
SJ18	IDLE	San Juan	-23.10	10.40	3.20
SJ19	IDLE	San Juan	-21.98	10.64	3.26
SJ20	IDLE	San Juan	-22.68	10.51	3.34
SJ13	IDLE	San Juan	-20.99	11.88	3.15
SJ9	IDLE	San Juan	-22.53	9.95	3.17
G7	IM/FI	Grand Jason	-18.67	15.32	3.23
F7	IM/FI	Grand Jason	-22.54	11.28	3.26
E7	IM/FI	Grand Jason	-19.37	16.20	3.13
D7	IM/FI	Grand Jason	-18.90	15.41	3.14
C7	IM/FI	Grand Jason	-17.66	16.34	3.13
B7	IM/FI	Grand Jason	-19.51	15.67	3.12
A7	IM/FI	Grand Jason	-19.36	15.21	3.16
C9	IM/FI	Rookery Valley	-18.83	11.50	3.17
B9	IM/FI	Rookery Valley	-20.05	11.07	3.17
G9	IM/FI	Rookery Valley	-19.06	15.19	3.19
F9	IM/FI	Rookery Valley	-18.87	12.47	3.14
E9	IM/FI	Rookery Valley	-20.97	10.53	3.36
D9	IM/FI	Rookery Valley	-15.68	14.60	3.14
B8	IM/FI	Sea Lion Island	-21.08	11.35	3.15
F8	IM/FI	Sea Lion Island	-20.47	11.03	3.19
E8	IM/FI	Sea Lion Island	-19.06	15.50	3.21
D8	IM/FI	Sea Lion Island	-18.71	13.57	3.19
C8	IM/FI	Sea Lion Island	-22.20	11.53	3.20

Supplementary Table 4. Analysis of Molecular Variance within each cluster (**Datasets**: **NORTH** and **SOUTH**)

AMOVA NORTH

	Df	Sum Sq	Mean Sq	Sigma	%	p
Between Pop	1	832	832	4.65	0.68	0.259
Between Subpop Within Pop	2	1405	702	0.97	0.14	0.645
Between samples Within Subpop	22	15284	695	15.36	2.24	0.267
Within samples	26	17264	664	664.02	96.94	0.205
Total	51	34786	682	685.00	100	

AMOVA SOUTH

	Df	Sum Sq	Mean Sq	Sigma	%	p
Between Pop	1	507	507	1.03	0.24	0.317
Between Subpop Within Pop	1	476	476	0.64	0.15	0.194
Between samples Within Subpop	78	34330	440	6.55	1.50	0.085
Within samples	81	34590	427	427.03	98.11	0.201
Total	161	69903	434	435.25	100	
